# Supplementary material for: Design and implementation of a digital site-less clinical study of serial rapid antigen testing to identify asymptomatic SARS-CoV-2 infection
Source: J Clin Transl Sci. 2023 May 10;7(1):e120. doi: 10.1017/cts.2023.540 (PMC10260333; doi:10.1017/cts.2023.540)

Supplemental Figures:
Study Workflow for Test Us At Home

Supplemental Figure 1: Enrollment process

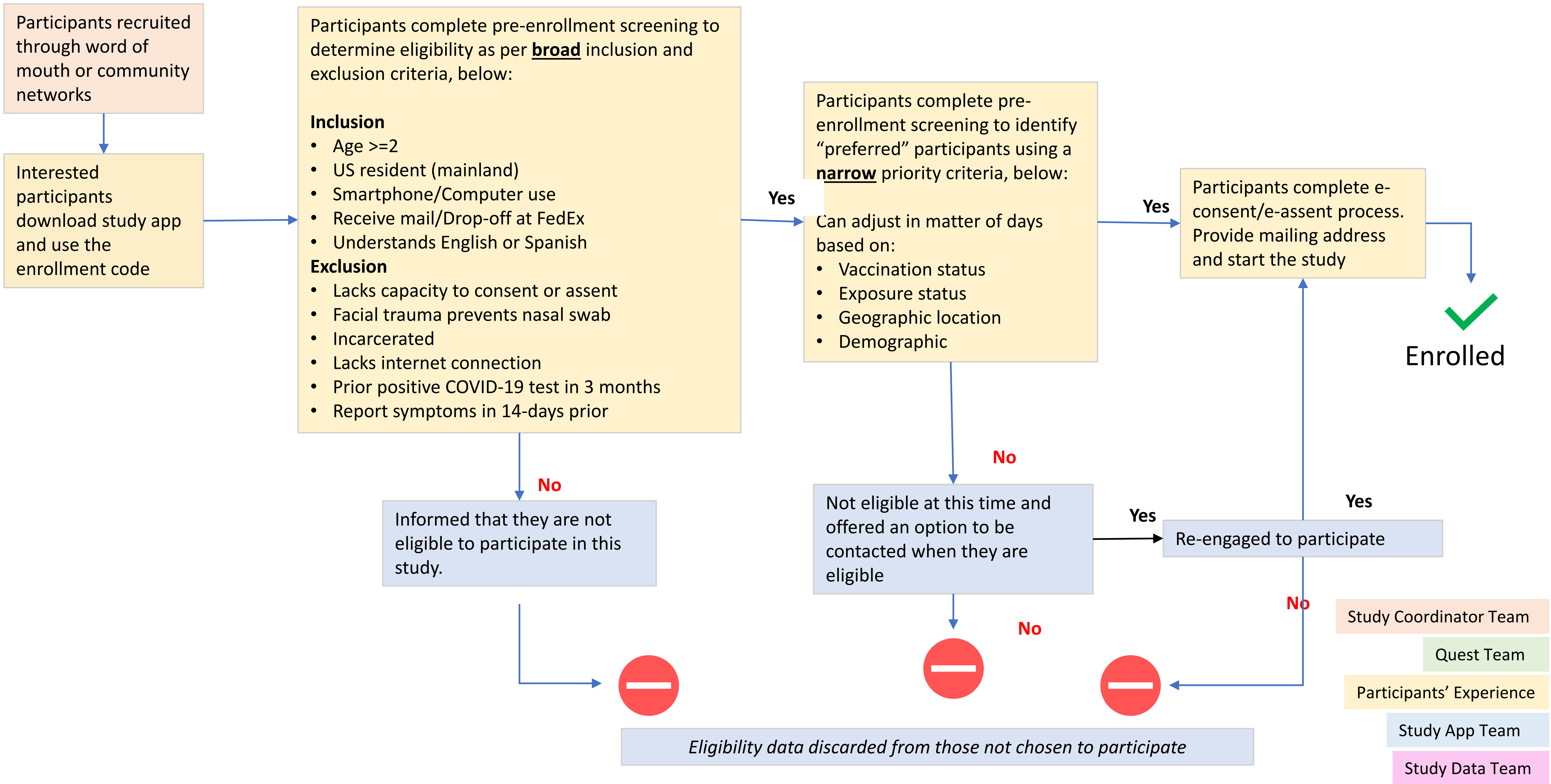

Supplemental Figure 2: Screenshot of Study App For Determining Preferred Status

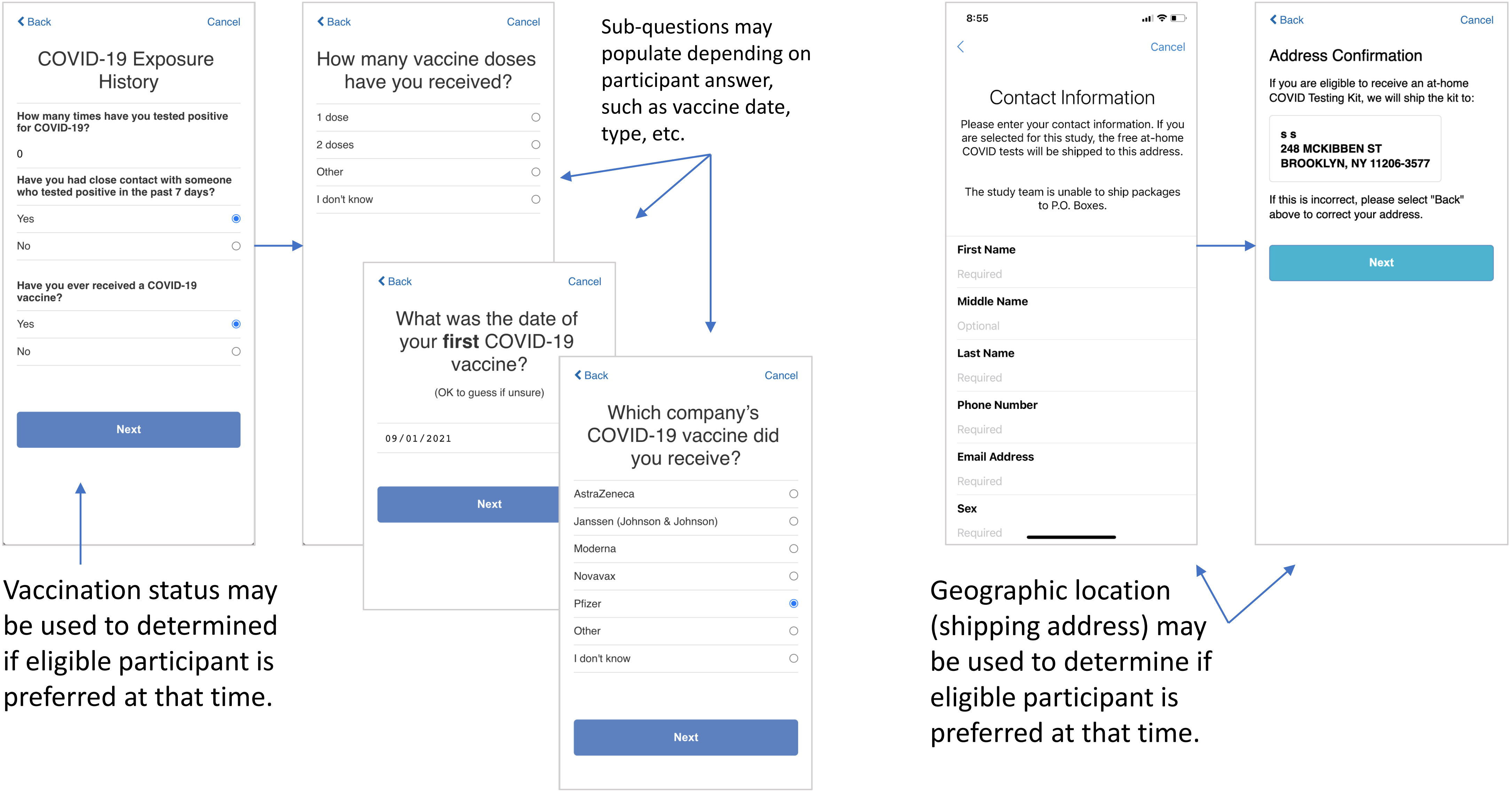

Depending on preferred status criteria at that time, participants will be enrolled or placed on waitlist (Supplemental Figure 3).

Supplemental Figure 3: Screenshot of Study App For Waitlisted and Preferred Participants

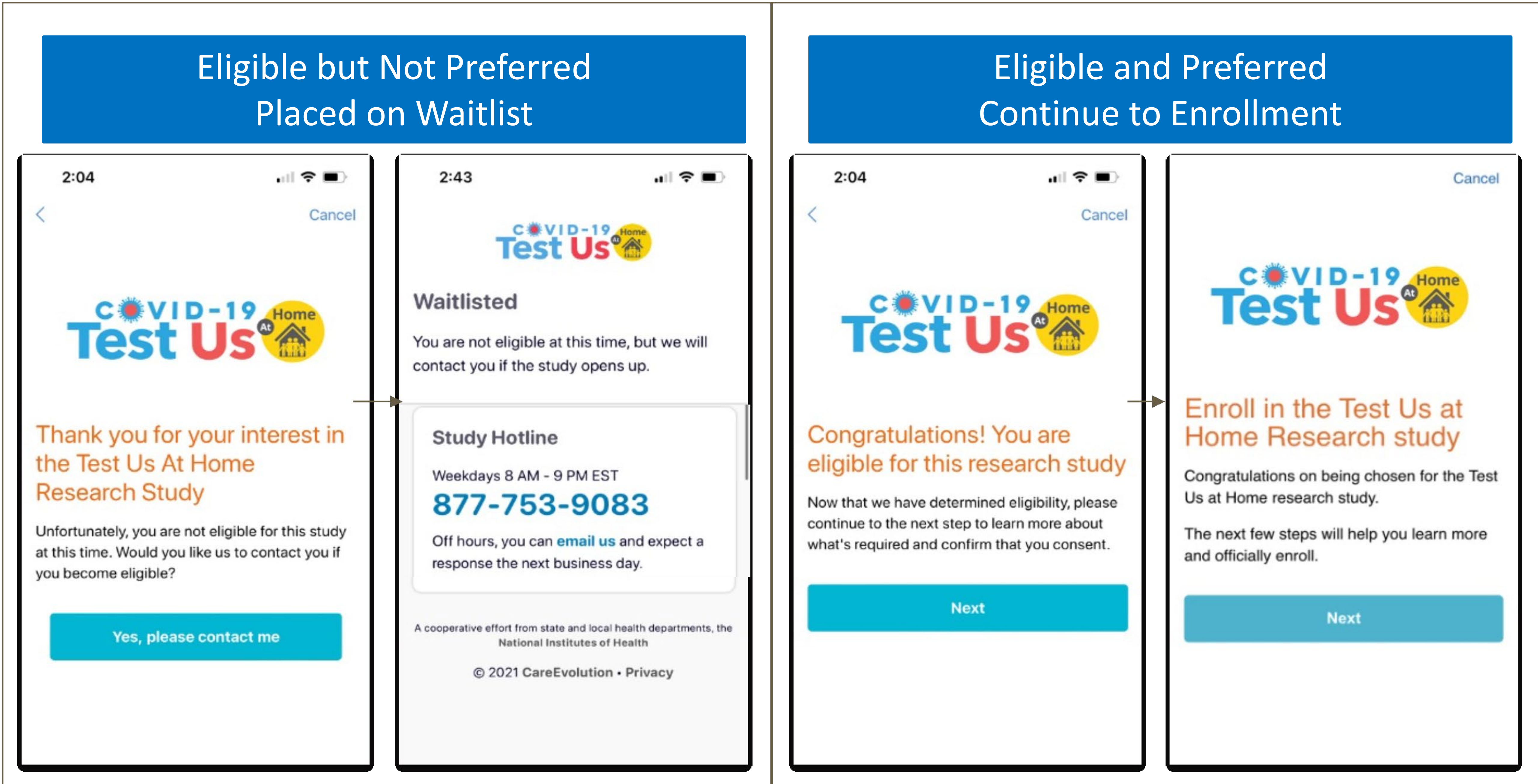

Supplemental Figure 4: Screenshots of Study App Consent Flow

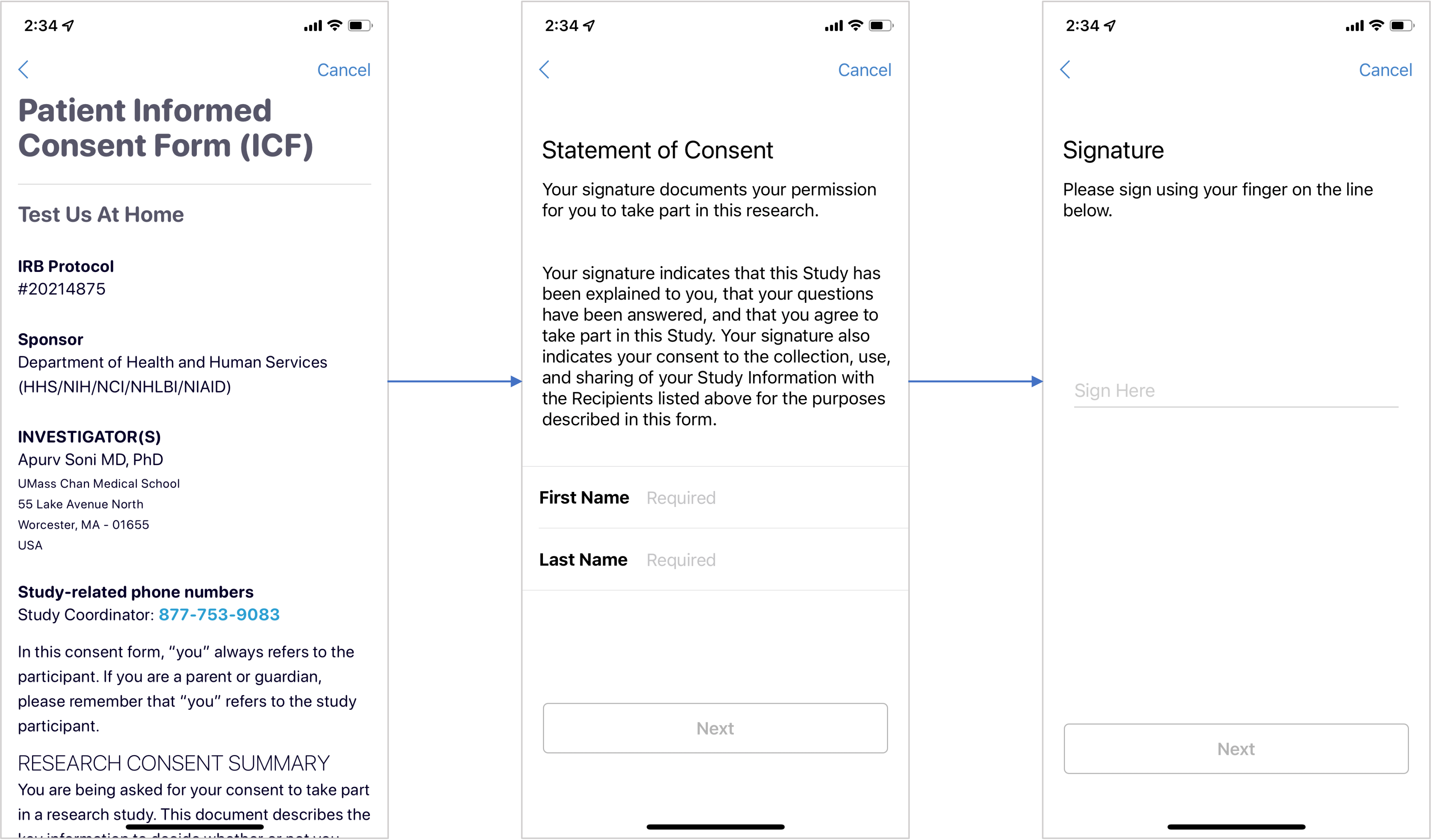

Supplemental Figure 5: Participant Compensation

Criteria	Amount
Participants complete 1 OTC test and 1 at home sample collection* on designated test day	$20 per test day (days 1- 13)
Participants complete at least 5 OTC tests and at home sample collections* and complete all of the study related activities^	$30 bonus
Participants complete all 8 OTC test and all 7 at home sample collections* and complete all of the study related activities^	$80 bonus

* To be eligible for reimbursement, participant must complete collection and shipping of the sample used for comparator testing (the Quest At Home Collection kit) on the same day of testing with the OTC test if the test is performed on Monday-Thursday; for tests performed on Friday-Sunday, participant must ship the sample on Monday to be eligible for reimbursement.

^ Study related activities include questionnaires, recording the OTC results, and for some OTC devices uploading an image of the test strip to the study app

Supplemental Figure 6: Test Assignment Strategy

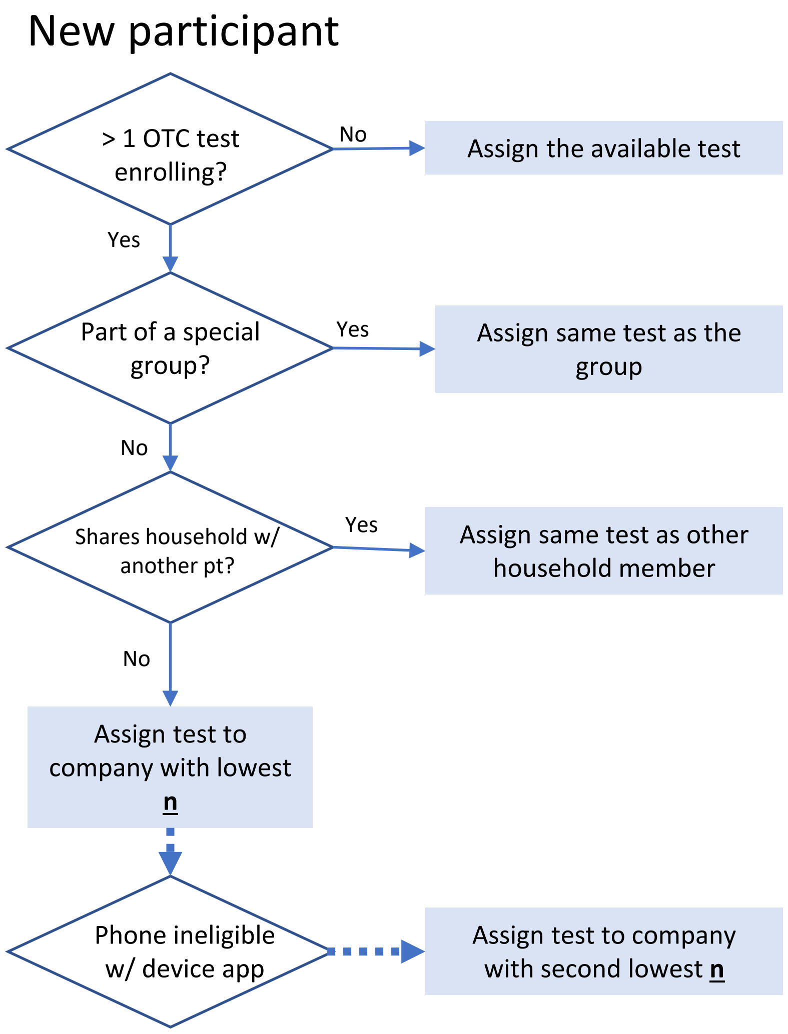

Rationale:

For the first week of enrollment, only one device was available. Up to three OTC tests available for OTC test assignment throughout the study.

In order to avoid a case-mix of tests in a specific group, assign participants with the same group code to the same OTC test assignment.

In order to avoid a case-mix of tests in a single household, assign participants with the same street address to the same OTC test assignment.

Algorithm allows the **n** to be based of the following parameters at different timepoints as needed:

- Enrolled
- Asymptomatic positives
- Participants that below 18
- Other characteristics

Study Coordinator Team

Quest Team

Participants' Experience

Study App Team

Study Data Team

Supplemental Figure 7: Quest Home Kit Orders and OTC Test Delivery

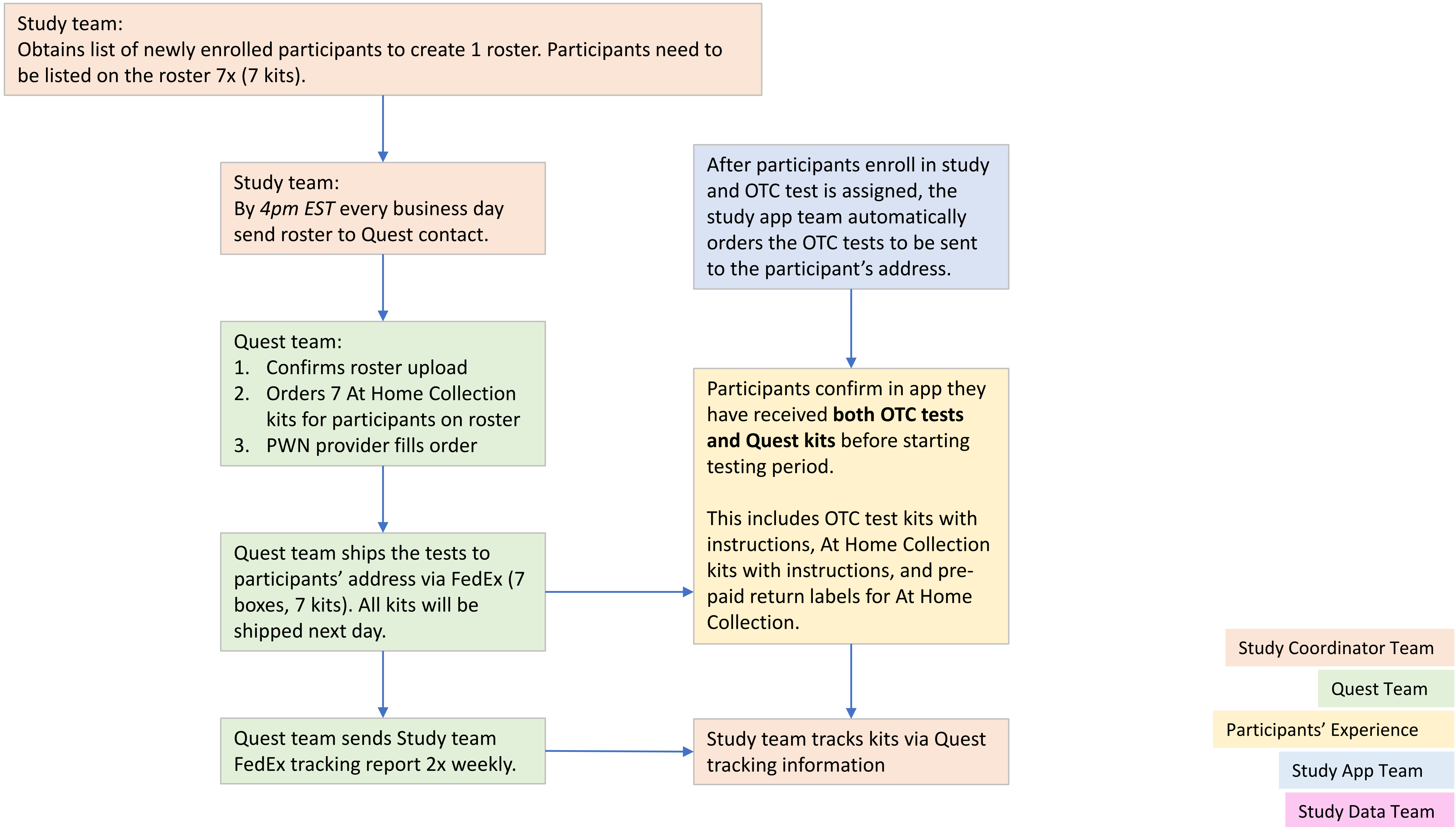

Supplemental Figure 8: Testing Period and Results

Participant confirms OTC and Quest materials received

15 Day Testing Period

Participants begin 15-day testing period with OTC and At Home Collection Kits according to testing schedule (Table 2).

Reminders will be sent to participants through the app on test days if test results have not been recorded yet on the designated test day.

If assigned OTC test requires, participants are prompted (with link) to download OTC device app and use as instructed.

1. Answer symptom questionnaire

2. Complete OTC test according to instructions. Input results in the Study app.

3. At Home Collection kit: That same day, wait at least 15min after taking the sample for the OTC test and then take sample for the At Home Collection Kit according to instructions.

Study team may reach out to participants if questionnaires, test results, or shipping samples are not completing on time.

Study team is available via hotline if participants have questions or issues while participating in the study

Report OTC test result in app; and asked to take a picture of the OTC test and upload in the app.

Participants receive phone call from study staff if OTC is reported as positive. Participants can reach study staff via hotline if they have any questions.

If testing on M-Th: schedule a pick-up at home or drop off in nearest FedEx drop box
If testing on F-Su: keep sample at room temp and ship on Mon via scheduled at home pickup or FedEx drop box

Quest team processes the sample collected at home.

Quest will report:
1. Any positive tests to the appropriate Health Dept.
2. All participant results through the Quest portal
3. All participant results back to the UMass Chan study team

Participants receive Quest test results 3-7 days after sample is shipped. Participants can reach study staff via hotline if they have any questions.

Study Day	1	2	3	4	5	6	7	8	9	10	11	12	13	14	15
Ag-RDT Device	X		X		X		X		X		X		X		X
Molecular Comparator	X		X		X		X		X		X		X		

X=Test completed on this day

Study Coordinator Team
Quest Team
Participants' Experience
Study App Team
Study Data Team

Supplemental Figure 9: Testing Process

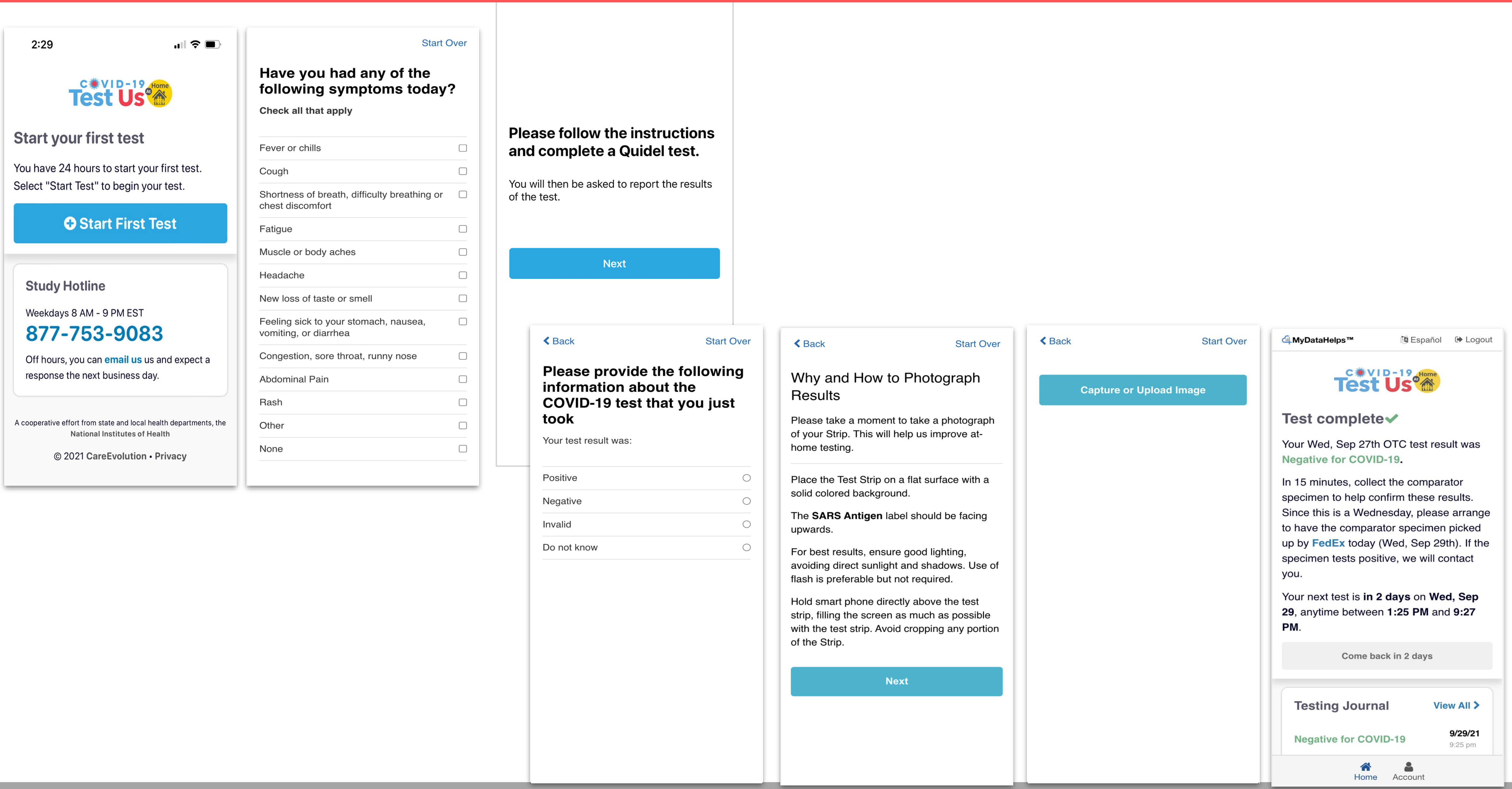

Supplemental Figure 10: Molecular Comparator Testing and Results

Participants follow testing schedule (Supplemental Figure 8) and ship At Home Collection kits back to Quest according to instructions.

Within 48 hours of receipt of sample, Quest:

1. Runs sample on Roche Cobas 6800 SARS-CoV-2 PCR and Quest Laboratory Developed RT-PCR (LDT)
2. Reports Roche Cobas results to participant via MyQuest portal & Dept. of Health.
3. Roche Cobas and Quest LDT results appear in study specific Quest data stream

Study team reviews Roche Cobas and Quest LDT test results via Quest data feed & orders tiebreaker on Hologic Aptima Transcription Mediated Amplification (TMA) if Roche Cobas and Quest LDT are discordant.

1. Quest runs tiebreakers on Hologic Aptima
2. All test results (Roche Cobas, Quest LDT, Hologic Aptima) appear in study-specific data stream for UMass Data Team.
3. Quest sends twice weekly chain of custody FedEx tracking report for home collection kits.
4. Send remnant sample back to UMass Chan

Study team communicates positive PCR results on molecular comparator assays (2/3 positive assays results as positive)

Study Coordinator Team

Quest Team

Participants' Experience

Study App Team

Study Data Team

Supplemental Figure 11: Overview of Test Us At Home Data Flow

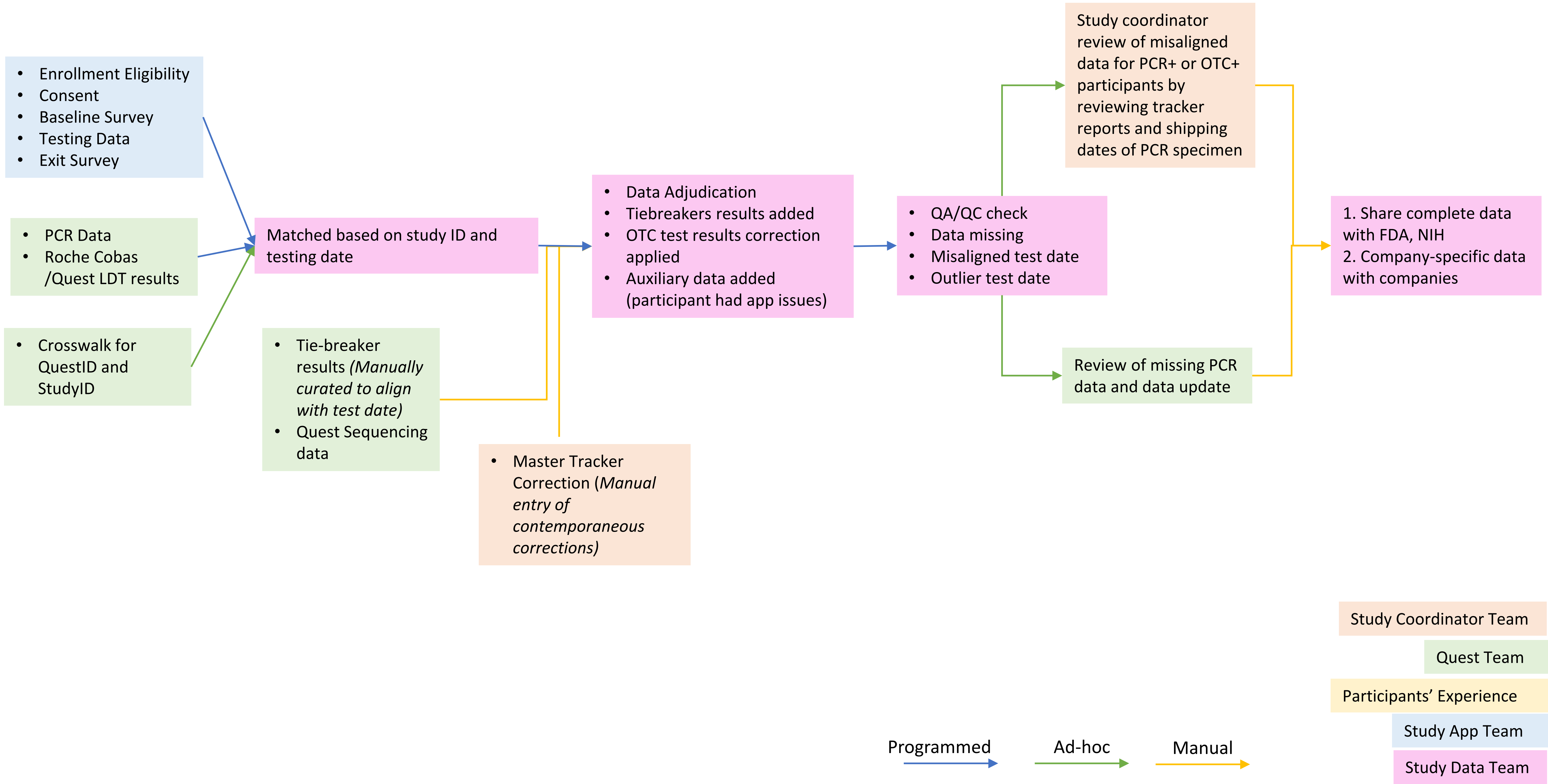

Supplement: Supplementary file 1 [file S205986612300540Xsup001.pdf]
